# Proposed Therapeutic Range of Treosulfan in Reduced Toxicity Pediatric Allogeneic Hematopoietic Stem Cell Transplant Conditioning: Results From a Prospective Trial

**DOI:** 10.1002/cpt.1715

**Published:** 2019-12-14

**Authors:** Robert Chiesa, Joseph F. Standing, Robert Winter, Zohreh Nademi, Jan Chu, Danielle Pinner, Frank Kloprogge, Susan McLellen, Persis J. Amrolia, Kanchan Rao, Giovanna Lucchini, Juliana Silva, Oana Ciocarlie, Arina Lazareva, Andrew R. Gennery, Bilyana Doncheva, Andrew J. Cant, Sophie Hambleton, Terence Flood, Elizabeth Rogerson, Kirsty Devine, Helen Prunty, Simon Heales, Paul Veys, Mary Slatter

**Affiliations:** ^1^ Bone Marrow Transplantation Department Great Ormond Street Hospital for Children NHS Foundation Trust London UK; ^2^ Pharmacy Department Great Ormond Street Hospital for Children NHS Foundation Trust London UK; ^3^ Infection, Immunity, and Inflammation Great Ormond Street Institute of Child Health University College London London UK; ^4^ Chemical Pathology Department Great Ormond Street Hospital for Children, NHS Foundation Trust London UK; ^5^ Bone Marrow Transplantation Department Great North Children's Hospital Newcastle upon Tyne Hospitals NHS Foundation Trust Newcastle upon Tyne UK; ^6^ Institute for Global Health University College London London UK; ^7^ Clinical Biochemistry Integrated Laboratory Medicine Directorate Newcastle upon Tyne Hospitals NHS Foundation Trust Newcastle upon Tyne UK

## Abstract

Treosulfan is given off‐label in pediatric allogeneic hematopoietic stem cell transplant. This study investigated treosulfan's pharmacokinetics (PKs), efficacy, and safety in a prospective trial. Pediatric patients (*n* = 87) receiving treosulfan‐fludarabine conditioning were followed for at least 1 year posttransplant. PKs were described with a two‐compartment model. During follow‐up, 11 of 87 patients died and 12 of 87 patients had low engraftment (≤ 20% myeloid chimerism). For each increase in treosulfan area under the curve from zero to infinity (AUC_(0‐∞)_) of 1,000 mg hour/L the hazard ratio (95% confidence interval) for mortality increase was 1.46 (1.23–1.74), and the hazard ratio for low engraftment was 0.61 (0.36–1.04). A cumulative AUC_(0‐∞)_ of 4,800 mg hour/L maximized the probability of success (> 20% engraftment and no mortality) at 82%. Probability of success with AUC_(0‐∞)_ between 80% and 125% of this target were 78% and 79%. Measuring PK at the first dose and individualizing the third dose may be required in nonmalignant disease.

**Study Highlights**

**WHAT IS THE CURRENT KNOWLEDGE ON THE TOPIC?**

☑ Unlike busulfan, it is thought that treosulfan does not require dose individualization by therapeutic drug monitoring (TDM) in pediatric allogeneic hematopoietic stem cell transplant (allo‐HSCT). A recent study, finding increased mortality with increased treosulfan area under the curve from zero to infinity (AUC_(0‐∞)_). Another including a heterogeneous group of diagnoses and conditioning regimens found no trend.

**WHAT QUESTION DID THIS STUDY ADDRESS?**

☑ Pharmacokinetic (PK) and long‐term allo‐HSCT outcome were studied in children receiving treosulfan‐fludarabine conditioning. The questions were: What are the PKs of treosulfan in infants and children? What is the relationship between treosulfan PK (AUC_(0‐∞)_) and mortality and donor engraftment?

**WHAT DOES THIS STUDY ADD TO OUR KNOWLEDGE?**

☑ Treosulfan AUC_(0‐∞)_ was strongly associated with mortality (high AUC_(0‐∞)_), and to a lesser extent poor engraftment (low AUC_(0‐∞)_). A target treosulfan AUC_(0‐∞)_ of 4,800 mg hour/L was defined. Interoccasion variability on clearance was low.

**HOW MIGHT THIS CHANGE CLINICAL PHARMACOLOGY OR TRANSLATIONAL SCIENCE?**

☑ TDM‐guided treosulfan dose individualization should be considered in infants and children undergoing allo‐HSCT for nonmalignant conditions.

Allogeneic hematopoietic stem cell transplantation (allo‐HSCT) is used in children for relapsed malignancies and nonmalignant conditions, such as primary immune deficiency.^1^ To deplete host immune cells and facilitate donor engraftment, children usually receive conditioning consisting of combination cytotoxic chemotherapy. Conditioning regimen intensity varies depending on the disease being treated. Malignant conditions tend to be treated with high intensity myeloablation, whereas nonmalignant conditions may be treated with lower intensity (lower dosing and/or fewer agents). Nevertheless, even with reduced toxicity conditioning, transplant‐related morbidity and mortality remain significant.^1^, ^2^, ^3^, ^4^, ^5^, ^6^, ^7^, ^8^, ^9^

Busulfan is commonly used in allo‐HSCT conditioning and studies have demonstrated therapeutic drug monitoring (TDM) and dose adjustment are associated with reduced transplant‐related mortality.^10^, ^11^, ^12^ The target area under the curve (AUC) and therapeutic range of busulfan was recently revised in a pharmacokinetic/pharmacodynamic (PK/PD) meta‐analysis and methods for personalizing exposure by measuring busulfan PK after the first dose and adjusting later dose(s) is now well established.^10^

Treosulfan is a busulfan analogue but, although busulfan causes direct DNA alkylation, treosulfan is a prodrug with alkylating activity mediated by its main epoxybutane derivatives.^13^ Since the first report of treosulfan‐based conditioning in pediatric allo‐HSCT in 2002, it has been increasingly used off‐label in children, largely due to a perceived wider therapeutic index and a lower propensity to cause veno‐occlusive disease/sinusoidal obstruction syndrome than busulfan.^14^ Data on treosulfan PK and toxicity in childhood are limited to mainly observational or retrospective studies,^15^, ^16^, ^17^, ^18^, ^19^ meaning the therapeutic range in this population is poorly defined.

This study aimed to characterize the PK/PD profile of treosulfan in children undergoing allo‐HSCT in an investigator‐initiated, multicenter phase II clinical trial. The primary end point was to measure treosulfan PK and the secondary end point was to assess its association with short‐term toxicity, graft failure, and mortality.

## Methods

### Ethics and patient recruitment

This was a prospective, open‐label, phase II study (ClinicalTrials.gov, NCT02048800; EudraCT number 2013‐003257‐20) conducted in accordance with the principles of Good Clinical Practice and the Declaration of Helsinki. Patients aged 28 days to 18 years old were eligible if they were scheduled to receive treosulfan‐fludarabine conditioning prior to allo‐HSCT. Patients and/or their legal guardians were asked to provide written informed consent and assent where appropriate at two centers in the United Kingdom: the Bone Marrow Transplant Department in Great Ormond Street Hospital, London, and the Bone Marrow Transplant Department in Great North Children's Hospital, Newcastle upon Tyne.

The study was split into an initial pilot phase and the main trial. In the pilot phase, PK sampling was undertaken following the first dose, whereas in the main study PK samples were taken after the first and third dose and detailed study of short‐term toxicity was performed. All patients were followed up for at least 1 year for survival and engraftment. Around 50 patients are required to capture important covariate effects in PK studies^20^ so we aimed to recruit at least 50 to the main study.

### Study conditioning regimen

The chemotherapy protocol consisted of treosulfan and fludarabine for all patients. Treosulfan was administered by 2‐hour i.v. infusion on days ‐7, ‐6, and ‐5 prior to allo‐HSCT at a total dose of 42 g/m^2^ (14 g/m^2^/dose) in children aged > 12 months, 36 g/m^2^ (12 g/m^2^/dose) in children aged 3–12 months, and 30 g/m^2^ (10 g/m^2^/dose) in children ≤ 3 months. Fludarabine was given from day ‐7 to day ‐3 prior to allo‐HSCT, at a total dose of 150 mg/m^2^. *In vivo* T‐cell depletion with alemtuzumab or antithymocyte globulin (ATG) was administered according to donor type and stem cell source. Further details of transplant procedures are given in the **Supplementary Materials**.

### Toxicity monitoring

In addition to mortality, in the main study, acute transplant‐related toxicity was assessed up to 1 month post‐allo‐HSCT, graded according to the National Cancer Institute Common Terminology Criteria for Advance Events criteria.^21^

The diagnosis of acute graft‐vs.‐host disease (GVHD) was made clinically and confirmed pathologically with skin, mucosal, or liver biopsy whenever possible. Grading of acute GVHD was performed according to the Seattle criteria.^22^ Chronic GVHD was assessed and scored according to the National Institute of Health (NIH) criteria.^23^

### Blood sample collection and treosulfan determination

Patients had indwelling multilumen central venous catheters *in situ*. Treosulfan was administered over 2 hours down one lumen and the line flushed. Following the end of the flush, blood was taken from a different lumen of the central venous catheter. A minimum of 3 mL of dead space blood was drawn and discarded prior to sampling. Initially, samples were drawn at the following times after completion of the flush postinfusion: 5, 15, and 30 minutes, and 1, 2, 3, 4, and 8 hours after the end of the infusion. To limit invasiveness and after confirming PK parameters (in particular AUC_(0‐∞)_) could still be estimated, an interim analysis showed sampling could be reduced to 4 postdose samples at: end of infusion, and 1, 2, and 4 hours after the end of the infusion.

Treosulfan concentrations in plasma were determined using a validated reverse‐phase high‐performance liquid chromatography method with refractometric detection in the Chemical Pathology Laboratory at Great Ormond Street Hospital.^15^ Further details are given in the **Supplementary Materials**.

### PK model building

Parameters for both one‐compartment and two‐compartment models assuming linear or nonlinear (Michaelis‐Menten) elimination were estimated using nonlinear mixed effects modeling with NONMEM version 7.4, using the first order conditional estimation algorithm.^24^ Interindividual variability was tested for all parameters assuming a log‐normal distribution, and interoccasion variability was tested for clearance and central volume. The residual error included additive and proportional terms.

Allometric size scaling of clearance and volume terms were added *a priori*, and addition of a sigmoidal postmenstrual age maturation function tested.^25^ Biomarkers relating to hepatic function (bilirubin and ALT), renal function (serum creatinine), and blood pH were tested on clearance. These covariates entered the model in the following form:

$$p_{i}=\theta_{p}{\left(\frac{c_{i}}{\bar{c}}\right)}^{\theta_{c}}$$

where *p_i_* is the individual parameter of interest, *c_i_* is the individual value of the covariate and $\bar{c}$ is the typical value of the covariate in the population. In the fixed allometric weight scaling, $c_{i}$ was the individual body weight, $\bar{c}$ was set to 70 kg, and $\theta_{c}$ was 0.75 for clearance and intercompartmental clearance, and 1 for central and peripheral volume. For bilirubin, ALT and pH, $\bar{c}$ was set to the median observed value, whereas for serum creatinine (because it is known to change with age) $\bar{c}$ was set to the median expected for age, as reported by Ceriotti *et al.* 26 In adolescents aged 15–18, a sex‐specific linear extrapolation was used to link the end of the Ceriotti *et al.* 26 function and the adult expected values, as previously reported by Johansson *et al.* 27 The sigmoidal age function scaling clearance used postmenstrual age (assumed a gestational age of 40 weeks when this was unavailable) and contained two estimated parameters, so the model took the following form:

$$p_{i}=\theta_{p}\frac{1}{1+{\left(\theta_{a}/a_{i}\right)}^{\theta_{\gamma }}}$$

where $a_{i}$ is an individual's postmenstrual age in weeks, $\theta_{a}$ is the age at which clearance is 50% mature, and $\theta_{\gamma }$ is a shape parameter.

The following categorical covariates were also tested: use of T‐cell depletion in the conditioning regime, whether patients were in the pilot or the main study, and study site. These categorical covariates entered the model as follows:

$$p_{i}=\theta_{p}\left(1+\theta_{c}I\right)$$

with *I* the indicator taking values of 1 when the covariate is present, and zero otherwise, and $\theta_{c}$ now being the fractional parameter change in the presence of the covariate, and allowed to take values of ≥−1.

For nested models, significance of the additional parameters was evaluated with the likelihood ratio test, the difference in ‐2 log‐likelihood (objective function value (OFV) in NONMEM) of the models being asymptotically $\chi_{1}^{2}$ distributed. Covariates were added if the likelihood ratio test indicated a significant improvement in fit at the level of *P* < 0.01. Further model evaluation consisted of plotting predictions vs. observations, and standardized residuals vs. time and predictions, a visual predictive check (1,000 samples) and a nonparametric bootstrap (1,000 samples). A cumulative AUC_(0‐∞)_ calculated from the sum of all three doses administered divided by the individual clearance estimate was generated for each patient.

### Statistical analysis of PDs

Two Cox proportional hazard survival analyses were performed in R, one to assess time to graft failure (chimerism in myeloid engraftment ≤ 20%) and one to assess time to mortality. Covariates considered were: cumulative treosulfan AUC_(0‐∞)_ for the three doses, age, use of T‐cell depletion (alemtuzumab or ATG) in conditioning, donor source and matching, diagnosis, and CD34 dose. Univariable analysis was performed, and if two or more were significant (*P* < 0.05) these were taken forward to a multivariable analysis.

Upon finding low AUC_(0‐∞)_ to be associated with engraftment ≤ 20%, and high AUC_(0‐∞)_ to be associated with mortality, a therapeutic target was derived by fitting a quadratic model of cumulative AUC_(0‐∞)_ vs. probability of success (defined as being alive at last follow‐up, with a myeloid engraftment > 20%). The linear predictor was defined as follows:

$$\rho =\beta_{0}+\beta_{1}\log\text{AUC}+\beta_{2}\log{\text{AUC}}^{2}$$

where logAUC was the natural logarithm of cumulative AUC_(0‐∞)_. This model was fitted to the binomial probability of success (defined as engraftment > 20% and being alive) with a generalized linear model in R. Logit, probit, and complimentary log–log canonical link functions were tested, the model with the lowest Akaike Information Criteria being chosen. Target concentration was defined as the logAUC, which maximized the probability of success, which by differentiating the expression above, yields:

$${\text{AUC}}_{\max}=\frac{\beta_{1}}{2\beta_{2}}$$

where AUC_max_ is the natural logarithm of cumulative AUC_(0‐∞)_, which maximizes probability of success. A nonparametric bootstrap with 10,000 samples was used to derive a 95% confidence interval (CI) on AUC_max_.

In the main study, an analysis investigating the relationship between cumulative AUC_(0‐∞)_ and National Cancer Institute (NCI) common toxicity criteria grade (0–5) for all major toxicity types was undertaken. The relationship with AUC_(0‐∞)_ and NCI grade was analyzed using the Kruskal–Wallis test by rank.

### Dosing simulations

During the course of our study, the company manufacturing treosulfan (Medac) suggested the following body surface area (BSA)‐based dosing scheme: 42 g/m^2^, reduced to 36 g/m^2^ for those with a BSA < 1 m^2^, and 30 g/m^2^ for those with a BSA < 0.5 m^2^. Ten thousand hypothetical patient characteristics (age 1 month–16 years) were generated using a published weight for age model^28^ and each was randomly assigned a serum creatinine value based on their age and sex by sampling from the model by Ceriotti *et al.* 26 Using the PK model, the cumulative AUC_(0‐∞)_ was simulated for our dosing scheme, the Medac scheme and doses derived from our final model accounting for age, weight, and serum creatinine, or only age and weight. These were compared with the target concentration derived from the quadratic model.

## Results

### Patients, donors, and transplant characteristics

A total of 87 children (30 in the pilot phase and 57 in the main study) receiving treosulfan as the sole alkylating agent in conditioning for allo‐HSCT between January 2013 and December 2016 were enrolled and followed up for at least 1 year posttransplant. The median follow‐up was 16 months (range 1247 months for surviving patients), baseline characteristics are detailed in **Table** 1. A total of 633 PK samples were obtained following the first and third doses, and no sample was below the assay lower limit of quantification. A total of 10 patients underwent the full PK sampling schedule (8 postdose samples), the remaining patients contributing 4 PK samples per occasion following the prespecified interim PK analysis.

**Table 1:** Patient characteristics

Characteristics	All patients	Died	Poor engraftment (< 20%)	Pilot study	Main study
Number of patients	87	11	12	30	57
Median age, months, at transplant (range)	19 (2–200)	18 (4–121)	24 (7–182)	39 (2–200)	16 (2–195)
Median weight, kg	10 (4.3–55.5)	10 (4.74–40)	11 (5.12–44.3)	13 (4.3–55.5)	10 (4.4–54.3)
Diagnosis					
Primary immune deficiency	79/87 (91%)	9/11 (82%)	12/12 (100%)	24/30 (80%)	55/57 (96%)
Inflammatory bowel disorder	5/87 (6%)	2/11 (18%)	0/12 (0%)	4/30 (13%)	1/57 (2%)
Juvenile myelomonocytic leukemia	2/87 (2%)	0/11 (0%)	0/12 (0%)	2/30 (7%)	0/57 (0%)
Inborn error of metabolism	1/87 (1%)	0/11 (0%)	0/12 (0%)	0/30 (0%)	1/57 (2%)
Donor type					
MSD	12/85 (14%)	1/9 (11%)	2/11 (18%)	8/29 (28%)	4/56 (7%)
MFD	4/85 (5%)	1/9 (11%)	1/11 (9%)	2/29 (7%)	2/56 (4%)
MUD	52/85 (61%)	2/9 (22%)	8/11 (73%)	11/29 (38%)	41/56 (73%)
MMUD	15/85 (18%)	4/9 (44%)	0/11 (0%)	8/29 (28%)	7/56 (12%)
MMFD	2/85 (2%)	1/9 (11%)	1/11 (9%)	0/29 (0%)	2/56 (4%)
Stem cell source					
Peripheral blood	53/85 (62%)	4/9 (44%)	7/11 (64%)	12/29 (41%)	41/56 (73%)
Bone marrow	22/85 (26%)	4/9 (44%)	4/11 (36%)	14/29 (48%)	8/56 (14%)
Umbilical cord blood	10/85 (12%)	1/9 (11%)	1/11 (9%)	3/29 (10%)	7/56 (12%)
Median CD34 + cell dose × 10^6^/kg (range)	11.7 (0.04–87)	7.5 (0.37–87)	8.9 (0.5–21.7)	7.5 (0.21–87)	13.5 (0.04–50.86)
Conditioning regimen					
Treosulfan + fludarabine	87/87 (100%)	11/11 (100%)	12/12 (100%)	30/30 (100%)	57/57 (100%)
Treosulfan dose					
30 g/m^2^	4/87 (5%)	0/11 (0%)	0/12 (0%)	1/30 (3%)	3/57 (5%)
36 g/m^2^	23/87 (26%)	4/11 (36%)	3/12 (25%)	7/30 (23%)	16/57 (28%)
42 g/m^2^	60/87 (69%)	7/11 (64%)	9/12 (75%)	22/30 (73%)	38/57 (67%)
*In vivo* T‐cell depletion					
Alemtuzumab	76/87 (87%)	10/11 (91%)	11/12 (92%)	25/30 (83%)	51/57 (89%)
Antithymocyte globulin	1/87 (1%)	0/11 (0%)	0/12 (0%)	0/30 (0%)	1/57 (2%)
GVHD prophylaxis					
Ciclosporin + mycophenolate	84/85 (99%)	9/9 (100%)	12/11 (109%)	29/29 (100%)	55/56 (98%)
Ciclosporin	1/85 (1%)	0/9 (0%)	0/11 (0%)	0/29 (0%)	1/56 (2%)

GVHD, graft‐vs.‐host disease; MFD, matched family donor; MMFD, mismatched family donor; MMUD, mismatched unrelated donor; MSD, matched sibling donor; MUD, matched unrelated donor.

### PK modeling

Treosulfan was given once daily for 3 days with a cumulative dose of 42 g/m^2^ (14 g/m^2^/dose) in children aged > 12 months, 36 g/m^2^ (12 g/m^2^/dose) in children aged 3–12 months, and 30 g/m^2^ (10 g/m^2^/dose) in children ≤ 3 months. The corresponding cumulative median (range) treosulfan AUC_(0‐∞)_ for the 3 doses was: 4,521 (4,352–4,740), 5,204 (2,321–9,023), and 4,590 (2,880–14,647) mg hour/L for the 4, 23, and 60 patients receiving these doses.

**Figure 1:**
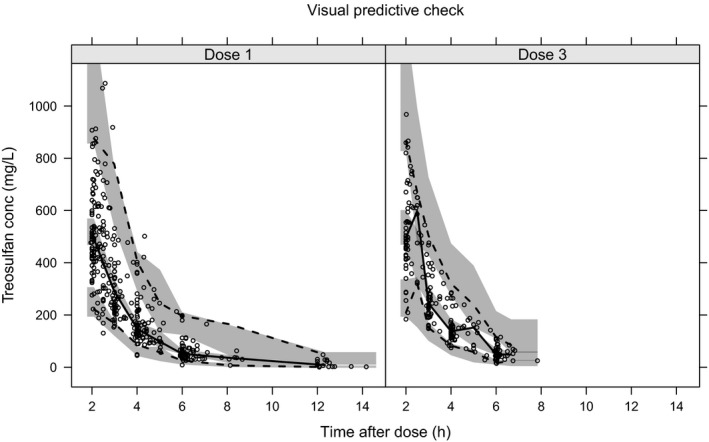
Visual predictive check of the final treosulfan pharmacokinetic model stratified for first and third doses. Shaded areas are the 95% confidence intervals of the 2.5th, 50th, and 97.5th percentiles of the model simulated data; lines are the corresponding percentiles of the raw data.

A two‐compartment model provided a superior fit to the one‐compartment (*P* < 0.01). The MichaelisMenten elimination did not result in successful minimization (Kaplan–Meier value became very large) or lower OFV, indicating linear clearance in the dose range studied. The addition of a sigmoidal maturation function decreased the OFV by 29 points. Serum creatinine was the only other covariate that significantly (*P* < 0.01) improved model fit. A scatter plot of correlations in the continuous covariates is given in **Figure** **S1**. **Table** 2 gives PK model parameters, a visual predictive check is given in **Figure** 1, and further goodness‐of‐fit and covariate plots are shown in the **Supplementary** **Figure** **S2**–**S4**. Parameter estimates are provided in **Table** 2.

**Table 2:** Pharmacokinetic model parameter estimates: All parameters being centered on a 70 kg individual using allometric scaling with exponents of 1 for volume terms and 0.75 for clearance terms

Parameter	Estimate (%RSE)	IIV %CV (%RSE)	IOV %CV (%RSE)	Bootstrap median (95% CI)	Bootstrap IIV %CV (95% CI)	Bootstrap IOV %CV (95% CI)
Pharmacokinetic model parameters						
CL (L/hour)	17.31 (5.6)	30% (25.1)	14% (49.8)	17.33 (15.38, 20.66)	30% (22, 37%)	13% (7, 18%)
V1 (L)	35.55 (4.7)	38% (27.1)	–	35.95 (30.54, 41.55)	38% (27, 47%)	–
Covariance of CL + V	–	0.95 (25.1)	–	–	0.943 (0.941, 0.947)	–
Q (L/hour)	9.36 (12.7)	–	–	8.99 (3.17, 13.13)	–	–
V2 (L)	9.89 (8.4)	43% (38.4)	–	9.51 (5.74, 11.9)	42% (20, 64%)	–
θ_a_ (postmenstrual age in weeks at 50% mature)	38.01 (4.6)	–	–	38.87 (28.17, 45.38)	–	–
θ_γ_ (shape parameter on age)	2.12 (3.2)	–	–	2.24 (0.79, 4.41)	–	–
θ_c_ (creatinine power)	−0.3 (30.7)	–	–	−0.31 (−0.49, −0.12)	–	–
Proportional error %	13.51 (0.2)	–	–	13.09 (10.07, 15.48)	–	–
Additive error (mg/L)	0.92 (61.6)	–	–	0.02 (0.01, 49.67)	–	–

Parameter	Estimate (%RSE)	95% CI	*P* value			
Quadratic model parameters						
β_0_	−138 (56%)	−310, −2.81	0.076			
β_1_	32.7 (55%)	1.27, 72.7	0.07			
β_2_	−1.9 (54%)	−4.25, −0.105	0.066			

Quadratic model parameter estimates (see Methods for description of parameters) with generalized linear model and complimentary log–log link function.

$\theta_{a}$, postmenstrual age in weeks to reach 50% of the mature value; $\theta_{c}$, allometric exponent of serum creatinine scaling for CL; $\theta_{\gamma }$, shape parameter in the maturation function; %CV, percentage of coefficient of variation; CI, confidence interval; CL, clearance; IIV, interindividual variability; IOV, interoccasion variability; Q, intercompartment clearance; V, volume; V1, central volume; V2, peripheral volume.

### Toxicity, survival, and engraftment

At last follow‐up, 76 of 87 children were alive. The causes of death from transplant‐related complications were: adenovirus infection (*n* = 3), Epstein–Barr virus‐related lymphoproliferative disease (*n* = 2), sepsis (*n* = 2), transplant‐associated micro‐angiopathy/veno‐occlusive disease (*n* = 1), multiorgan failure (*n* = 2), and progressive encephalopathy (*n* = 1).

**Figure 2:**
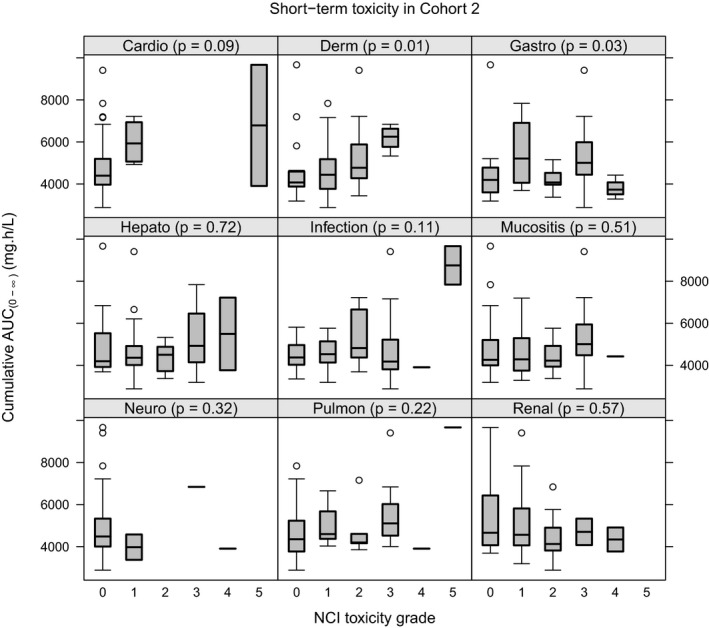
Short‐term toxicity National Cancer Institute (NCI) grade in the main study vs. cumulative area under the curve from zero to infinity (AUC_(0‐∞)_). Significance according to the Kruskal–Wallis test by rank shown in brackets.

**Figure** 2 summarizes organ toxicity within 30 days posttransplant in the main study (*n* = 57), graded according to the NCI criteria. Overall treosulfan was well tolerated, although gastrointestinal toxicity was common. Grade II and grade III–IV acute GVHD occurred in 24 patients (28%) and 3 patients (3%), respectively, with no strong relationship to treosulfan AUC_(0‐∞)_ (**Figure** **S5**). Two patients (2%) developed chronic GVHD.

Median neutrophil recovery time was 16 days (range 8–33 days). Median platelet recovery time was 12 days (range 5–101 days). Only 1 of 85 patients who received the allo‐HSCT presented primary engraftment failure. At last follow‐up, myeloid (CD15+ cells) donor engraftment was ≥ 95% in 52 children, 21–94% in 21 children, and ≤ 20% in 12 patients. Three patients had very poor donor engraftment (≤ 5%). T‐cell (CD3+ cells) donor engraftment was ≥ 95% in 57 children, 21–94% in 27 children, and ≤ 20% in 1 patient.

### PD modeling and dosing simulations

Survival and ≤ 20% engraftment were modeled in a stepwise manner using Cox proportional hazards. First, univariable analysis was performed and significant covariates (*P* < 0.05) taken forward to a multivariable analysis (**Table** 3). For mortality, two covariates were significant (AUC_(0‐∞)_) and being in receipt of a mismatched donor, whereas for engraftment there was a trend for low AUC_(0‐∞)_ to be associated with poor engraftment (hazard ratio (95% CI) = 0.61 (0.361.04); *P* = 0.072).

**Table 3:** **Univariable Cox proportional hazards model for mortality and engraftment**

Covariate	Mortality hazard ratio	Mortality *P* value	Engraftment hazard ratio	Engraftment *P* value
Cumulative treosulfan AUC_(0-∞)_ g hour/L	1.46 (1.23, 1.74)	0.000021	0.61 (0.36, 1.04)	0.072
Age, months	1 (0.98, 1.01)	0.50	1 (0.99, 1.01)	0.750
CD34 + dose (×10^6^/kg)	1.03 (0.99, 1.07)	0.16	0.95 (0.89, 1.02)	0.160
Stem cell source – BM	2.43 (0.61, 9.74)	0.21	2.13 (0.61, 7.43)	0.240
Stem cell source – UCB	1.33 (0.15, 11.97)	0.80	0.64 (0.08, 5.35)	0.680
Received ATG/alemtuzumab	1.72 (0.22, 13.5)	0.61	2.39 (0.3, 19)	0.410
Donor – MFD/MSD	3.27 (0.46, 23.25)	0.24	1.61 (0.42, 6.18)	0.490
Donor – MMFD/MMUD	8.98 (1.74, 46.42)	0.0088	0.45 (0.06, 3.6)	0.450
Diagnosis – not PID	2.61 (0.56, 12.12)	0.22	0 (0, Inf)	1.000

Multivariable analysis	Multivariable mortality hazard ratio	Multivariable mortality *P* value		
Cumulative treosulfan AUC_(0-∞)_ g hour/L	1.32 (1.07, 1.64)	0.0093		
Donor – MMFD/MMUD	3.35 (0.74, 15.23)	0.1200		

For the binary variables the result relates to a patient not receiving ATG or alemtuzumab with a matched unrelated donor, with peripheral blood stem cell source, and a diagnosis of primary immune deficiency.

ATG, antithymocyte globulin; AUC_(0‐∞)_, area under the curve from zero to infinity; BM, bone marrow; MFD, matched family donor; MMFD, mismatched family donor; MMUD, mismatched unrelated donor; MSD, matched sibling donor; PID, primary immune deficiency; UCB, umbilical cord blood.

**Figure 3:**
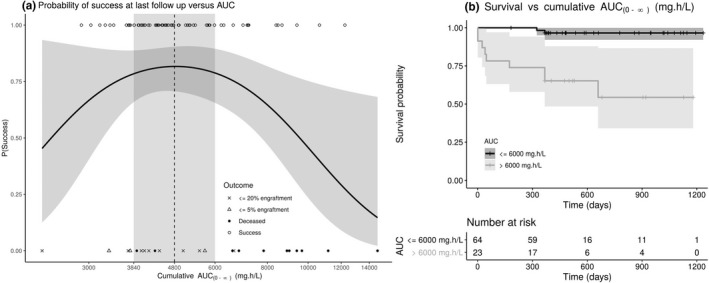
Left side: Pharmacodynamic model fit of the quadratic expression describing the change in probability of success (vertical axis) with increasing cumulative area under the curve from zero to infinity (AUC_(0‐∞)_) (horizontal axis). Black line and associated shaded area is the model fit and 95% confidence interval, open circles are AUC_(0‐∞)_ for patients with successful outcomes; crosses are for patients with ≤ 20% engraftment, triangles are for patients with < 5% engraftment, and black points are patients who died. Vertical dashed line gives AUC_(0‐∞)_ at which probability of success is maximized, vertical shaded area gives AUC_(0‐∞)_ region covering 80% probability of success. Right side: Kaplan–Meier curve for 12‐month overall survival in patients above and below the upper success probability AUC_(0‐∞)_ cutoff.

Upon finding low treosulfan AUC_(0‐∞)_ associated with poor engraftment and high treosulfan AUC_(0‐∞)_ with mortality, we modeled the probability of success (alive at last follow‐up, with a myeloid engraftment > 20%) with a quadratic generalized linear model in R (see Methods section). The lowest Akaike Information Criteria was found with the complimentary log‐log canonical link. The model fit is shown in **Figure** 3 **a** and parameter estimates are presented in **Table** 2. The probability of success was maximized at 82% for a treosulfan AUC_(0‐∞)_ of 4,829 mg hour/L (cumulative of the 3 doses). A nonparametric bootstrap revealed this estimate to be unbiased but imprecise with bootstrap median (95% CI) of 4,876 (1,623–10,839) mg hour/L. The target was, therefore, rounded to two significant figures to 4,800, which also gives an 82% probability of success, whereas the interval between 3,863 and 6,037 mg hour/L represents the treosulfan AUC_(0‐∞)_ interval suggested for narrow therapeutic index drugs,^29^ which gives corresponding probabilities of success of 78% and 79%, respectively.

In our study, only 57% of children achieved this AUC_(0‐∞)_ range, whereas 16% had a treosulfan AUC_(0‐∞)_ below the lower cutoff (3,840 mg hour/L) and 26% patients had a treosulfan AUC_(0‐∞)_ above the upper cutoff (6,000 mg hour/L).

Transplant‐related mortality in patients with treosulfan AUC_(0‐∞)_ > 6,000 mg hour/L was 39% compared with 3% in those with a treosulfan AUC_(0‐∞)_ < 6,000 mg hour/L. The corresponding survival was significantly lower (*P* < 0.0001) in patients with AUC_(0‐∞)_ values above and below this cutoff (**Figure** 3 **b**).

**Figure 4:**
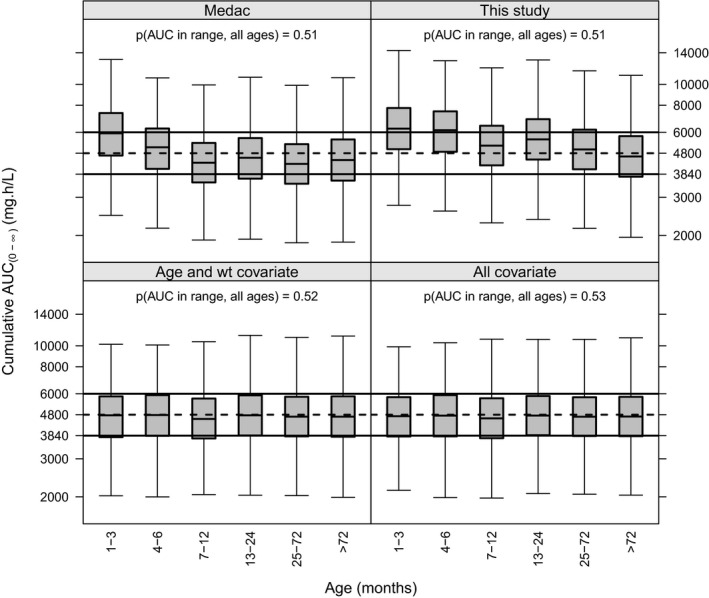
Simulated comparison of dosing used in our study against dosing proposed by Medac on cumulative area under the curve from zero to infinity (AUC_(0‐∞)_) with age. The lower two plots give target attainment if doses were based on the covariates in the pharmacokinetic model (either age and weight, or age, weight, and creatinine). Dashed horizontal lines give the upper and lower cumulative AUC_(0‐∞)_ targets with overall probability of target attainment printed on each plot.

Simulated treosulfan AUC_(0‐∞)_ for our dosing scheme (30, 36, and 42 g/m^2^ for patients < 3 months, 3–12 months, and > 12 months, respectively) and the Medac dosing scheme (30, 36, and 42 g/m^2^ for BSA ≤ 0.5, 0.5–1 and > 1 m^2^, respectively) are shown in **Figure** 4. In addition, calculating the dose from our model with and without the creatinine covariate is presented. This was achieved by taking the typical clearance for a patient based on their covariates and defining the dose to target a cumulative AUC_(0‐∞)_ of 4,800 mg hour/L as follows:

$$\text{Dose}=4800\times {\text{CL}}_{\text{PRED}}$$

where

$${\text{CL}}_{\text{PRED}}=17.31{\left(\frac{{\text{wt}}_{i}}{70}\right)}^{0.75}\frac{1}{1+{\left(38.01/a_{i}\right)}^{2.12}}{\left(\frac{\sec r_{i}}{\text{mscr}}\right)}^{-0.3}$$

with wt*_i_* being the individual's weight in kg, *a_i_* the postmenstrual age in weeks, secr*_i_* the individual's serum creatinine in µmol/L, and mscr is the median creatinine for age predicted from the Ceriotti model.^26^

## Discussion

In a prospective clinical trial of treosulfan PK in pediatric allo‐HSCT, treosulfan AUC_(0‐∞)_ was associated with poor donor engraftment and mortality. Because all but two patients in our study had nonmalignant disease, our findings should be inferred only to apply to this group. This has facilitated the proposal of a therapeutic target of cumulative AUC_(0‐∞)_ 4,800 mg hour/L. Being within 80–125% of this target cannot be met in ~ 50% of patients through dosing by covariates alone (**Figure** 4), hence, the major finding is that a TDM‐guided treosulfan dose adjustment should be explored.

Children with a cumulative treosulfan AUC_(0‐∞)_ > 6,000 mg hour/L had transplant‐related mortality of 39%, whereas patients with AUC_(0‐∞)_ below this had transplant‐related mortality of 3%. The only other significant relationship with mortality in the univariable analysis was receiving a mismatched donor, but upon multivariable analysis the strength of this association was reduced (**Table** 3). Our choice of using all transplant‐related mortality could be questioned given that three patients died beyond 100 days of viral‐associated complications. It could be argued that because treosulfan is mainly myeloablative, high AUC_(0‐∞)_ may not be related to these cases, and fludarabine, lymphocyte depletion, or the patient's underlying immune deficiency are more important. However, treosulfan does have broader immunosuppressive effects than simple myeloabaltion^30^ and its role in the establishment and longer term effects from viral complications cannot be completely ruled out. Truncating the survival analysis at 100 days and 6 months shows the effect is less strong early on but by 6 months is similar to the overall effect (100‐day hazard ratio (95% CI): 1.18 (0.93, 1.51); 6‐month hazard ratio: 1.3 (1.06, 1.6)).

Although an observational study of 77 children by van der Stoep *et al.* 19 did not find a correlation between treosulfan AUC_(0‐∞)_ and mortality, this may be due to the heterogeneity of conditioning as 67.5% patients also received thiotepa. In contrast, children enrolled in our prospective clinical trial received homogeneous conditioning of treosulfan and fludarabine only. Recently, an observational study in children undergoing treosulfan conditioning for allo‐HSCT in thalassemia major found a trend of 82% survival in patients with an AUC_(0‐∞)_ of < 5,484 mg hour/L compared with only 68% in patients above this threshold.^31^ Taken together with our result possibly indicates the need to individualize doses in patients with nonmalignant disease. To draw firm conclusions on causation a prospective study is required, because patients who enter the conditioning period with lower treosulfan clearance may have comorbidities predisposing them to mortality, which would not be prevented by lowering treosulfan AUC_(0‐∞)_.

The association between conditioning drug exposure and clinical outcome in pediatric allo‐HSCT has been explored in a number of studies. Busulfan studies have shown an association among exposure and toxicity and engraftment^32^, ^33^ with TDM and personalization utilized for a number of years. The US Food and Drug Administration (FDA) proposes a target of 900–1,350 µM minutes^34^ whereas the European Medicines Agency (EMA) proposes 900–1,500 µM minutes.^35^ Surprisingly, these targets are based on small observational studies.^11^, ^12^ A larger but retrospective study on 674 patients with malignant and nonmalignant conditions recently derived a higher target of 1,225–1,575 µM minutes.^10^ Likewise, Admiraal *et al.* 36 recently demonstrated that an optimal exposure to ATG is associated with higher event‐free survival and lower risk of acute GVHD in adults undergoing allo‐HSCT.

Early reports on treosulfan in pediatric allo‐HSCT by Glowka *et al.* 15 showed children receiving treosulfan demonstrated large variability in AUC_(0‐∞)_, suggesting that TDM may be needed. More recently, van der Stoep *et al.* 19 described treosulfan PK in 77 children undergoing allo‐HSCT, showing interindividual and interoccasion clearance variability of 33–56% and 13.9%, respectively. Our results (30% and 14%, respectively) are similar.

Our target was found to be rather imprecise (95% CI 1,623–10,839 mg hour/L) upon nonparametric bootstrap but the median (4,876 mg hour/L) was close to our estimate, suggesting it is unbiased. The imprecision is likely due to the small number of events but for now our data remain one of the largest to date. It has been proposed that an acceptable range is being within 80% and 125% of a target value, and if the log‐normal distribution is assumed this translates to 90% of patients achieving that range if unexplained variability is 13.6% coefficient of variance.^29^ Comparing our interindividual and interoccasion variability values on clearance shows dosing by covariates alone will not achieve this target (**Figure** 4), because the first dose AUC could be measured and the interoccasion variability is 14%, it is likely the cumulative AUC_(0‐∞)_ for the three doses could readily be targeted. Our future work will include a detailed optimal design and simulation‐estimation study to evaluate the potential of treosulfan TDM.

The clearance estimate in our model was scaled by both weight and age. Weight scaling used a fixed allometric model, which approximately follows BSA and for older children and has recently been shown to apply for most drug classes.^37^ Hence, BSA‐based dosing should give similar AUC_(0‐∞)_ for children older than around 2 years. It is also well known that in the first year of life BSA‐based dosing leads to higher AUC_(0‐∞)_ due to immaturity in clearance.^37^ There are a number of ways to model declining clearance with younger age and recently it was shown that most give equivalent results,^25^ and, hence, we used the standard method proposed by Holford *et al.* 38 The major benefit of using this standard method is that it is then very straightforward to compare clearance values between different studies. Our estimate of clearance was 17.31 L/hour/70 kg, which is similar to that in a recent treosulfan observational study (17.9 L/hour/70 kg),^19^ and the surface‐area scaled value in the recent thalassemia study (20.07 L/hour/1.73 m^2^),^31^ all of which are somewhat higher than the value recently estimated by Danielak *et al.* 18 (14.7 L/hour/70 kg). The likely reason is the latter study only included 15 patients with a wide age range, the youngest of whom was < 6 months old, yet no age‐related maturation term was used. Recently, a model‐based reanalysis of the data by van der Stoep *et al.* in 2017^19^ found a similar maturation half‐time to ours (38 weeks) with a lower shape parameter (1.2).^39^ It is likely our shape parameter is more reliable because our patients were, on average, 19 months old whereas in that study the median age was 52 months,^39^ and, furthermore, the busulfan maturation half time and the shape parameter were 40 weeks and 2.2 in a large meta‐analysis.^40^

A possible reason for decreased clearance in younger patients is immaturity in glomerular filtration rate because around 40% is excreted renally. During the covariate analysis we found serum creatinine to be inversely correlated with clearance. This was modeled by multiplying clearance by the ratio of serum creatinine to the age‐expected serum creatinine raised to an estimated power.^27^ Although this relationship was statistically significant and so retained in the model, the covariate power estimate of −0.3 means even in a child with a twofold higher than age‐expected creatinine, this would only decrease clearance by around 19%.

Target attainment through dosing by covariates was similar from each of the tested dosing regimens (text probabilities in **Figure** 4). Because the simulated population had a uniform distribution of ages, it seems that surface area or allometric dosing gives very similar target attainment in patients aged > 2 years. The differences come in the younger age groups where dosing by age and weight seems optimal, the addition of creatinine adding little. The Medac scheme showed a trend for reduced overexposure compared with dosing in our study. However, because all covariate‐based dosing gives target attainment of around 50%, TDM will still be required.

In conclusion, the PK of treosulfan in children have been characterized and an association with high AUC_(0‐∞)_ and mortality and low AUC_(0‐∞)_ and poor engraftment was found. A prospective study on TDM‐guided personalization is warranted.

## Funding

This study was funded by the Great Ormond Street Hospital Children's Charity. Support at the institution level came from the National Institute for Health Research Biomedical Research Centre at Great Ormond Street Hospital for Children NHS Foundation Trust and University College London. J.F.S. and F.K. were supported by United Kingdom Medical Research Council (MRC) Fellowships (Grants M008665 and P014534).

## Conflict of Interest

M.S. has received travel grants to attend meetings by Medac and honoraria for speaking engagements. A.G. has received travel grants to attend meetings by Medac. All other authors declared no competing interests for this work.

## Author Contributions

R.C., J.F.S., R.W., and M.S. wrote the manuscript. R.C., J.F.S., H.P., P.V., and M.S. designed the research. R.C., R.W., Z.N., J.C., D.P., S.M., P.J.A., K.R., G.L., J.S., O.C., A.L., A.R.G., B.D., A.J.C., S.H., T.F., E.R., K.D., P.V., and M.S. performed the research. R.C., J.F.S., and F.K. analyzed the data. H.P., R.W., and S.H. contributed new analytical tools.

## Supporting information

Supplementary Methods, Figures S1‐S7, Treosulfan NONMEM PK model code.
