# Supplementary material for: Proposed Therapeutic Range of Treosulfan in Reduced Toxicity Pediatric Allogeneic Hematopoietic Stem Cell Transplant Conditioning: Results From a Prospective Trial
Source: Clin Pharmacol Ther. 2019 Dec 14;108(2):264–73. doi: 10.1002/cpt.1715 (PMC7484914; doi:10.1002/cpt.1715)
Supplement: Supplementary file 1 — Supplementary Methods, Figures S1‐S7, Treosulfan NONMEM PK model code. [file CPT-108-264-s001.pdf]

SUPPLEMENTARY MATERIAL: Proposed therapeutic range of treosulfan in reduced toxicity pediatric allogeneic hematopoietic stem cell transplant conditioning: results from a prospective trial

Contents

1 Supplementary Methods **2**
- 1.1 Transplant procedures 2
- 1.2 Sample preparation and analysis of treosulfan concentrations 2

2 Correlations in continuous covariates **3**

3 Further pharmacokinetic results **4**
- 3.1 Treosulfan model basic goodness-of-fit 4
- 3.2 AUC and clearance relationship with age 5

4 Further Pharmacodynamic results **7**
- 4.1 Graft versus Host Disease and AUC 7
- 4.2 Neutrophil recovery and AUC 8
- 4.3 Platelet recovery and AUC 9

5 NONMEM pharmacokinetic model code **10**

1 Supplementary Methods

1.1 Transplant procedures

HLA typing was performed by molecular typing for HLA class I and II loci; mismatch was defined as anything < 9 out of 10 HLA identical. Graft *versus* host disease (GvHD) prophylaxis consisted of ciclosporin (CsA, from day -3 with doses adjusted to a trough whole blood level of 100-250 ng/mL with TDM) and mycophenolate mofetil (MMF, from day 0 to day +28, then weaned over 3 weeks in the absence of GvHD).

Antimicrobial prophylaxis consisted of either ciprofloxacin (from day -10, until neutrophil count - 1 x 10^9/L) or co-trimoxazole, aciclovir (from day -10, until 1 year post-transplant) and itraconazole or liposomal amphotericin B (from day -11, until neutrophil count - 1 x 10^9/L and no steroid treatment). Prophylaxis of *Pneumocystis jiroveci* pneumonia was with co-trimoxazole daily from day -10 to day -1 and then, after myeloid recovery, until a CD4+ T-cell count > 0.3 x 10^9/L and absence of chronic GvHD/immunosuppressive treatment or throughout the transplant period.

Myeloid recovery was defined as the first of 3 consecutive days with an absolute neutrophil count exceeding $0 \cdot 5$ x 10^9/L, while platelet recovery was defined as an unsupported (by transfusion) platelet count exceeding 20 x 10^9/L. Engraftment was monitored by short tandem repeat variability on peripheral blood in the T-cell (CD3+ cells) and myeloid (CD15+ cells) compartment.

1.2 Sample preparation and analysis of treosulfan concentrations

Blood samples were adjusted to a final pH of 5.5 by the addition of 50 µL of 1 M citric acid per 1 mL of blood immediately after collection to avoid artificial ex vivo degradation of treosulfan and then centrifuged to obtain plasma. The resulting plasma samples were frozen at -20°C until analysis.

Treosulfan concentrations in plasma were determined using a validated reverse-phase HPLC method with refractometric detection in the Chemical Pathology laboratory at Great Ormond Street Hospital. This method was validated in-house with a limit of quantitation of 10 µg/mL, within batch imprecision of 4.5% and inter-day imprecision of 7.5%. Plasma samples were removed from the freezer and allowed to come to room temperature over an hour. The 10 g/L sodium barbital ISTD was removed from the freezer and allowed to defrost over this time. From the sample 200 µL was taken by fixed volume pipette into Millipore centrifugal filter tubes (10,000 kDa cut off). This was followed by 25L ISTD and 25L distilled water. The tubes were briefly vortexed and then placed into a centrifuge and spun at 13,000 rpm for 15 minutes. The filtered solution was then transferred into UHPLC vials. The UHPLC system was set up at the beginning of the day to allow time for the baseline to stabilise.

2 Correlations in continuous covariates

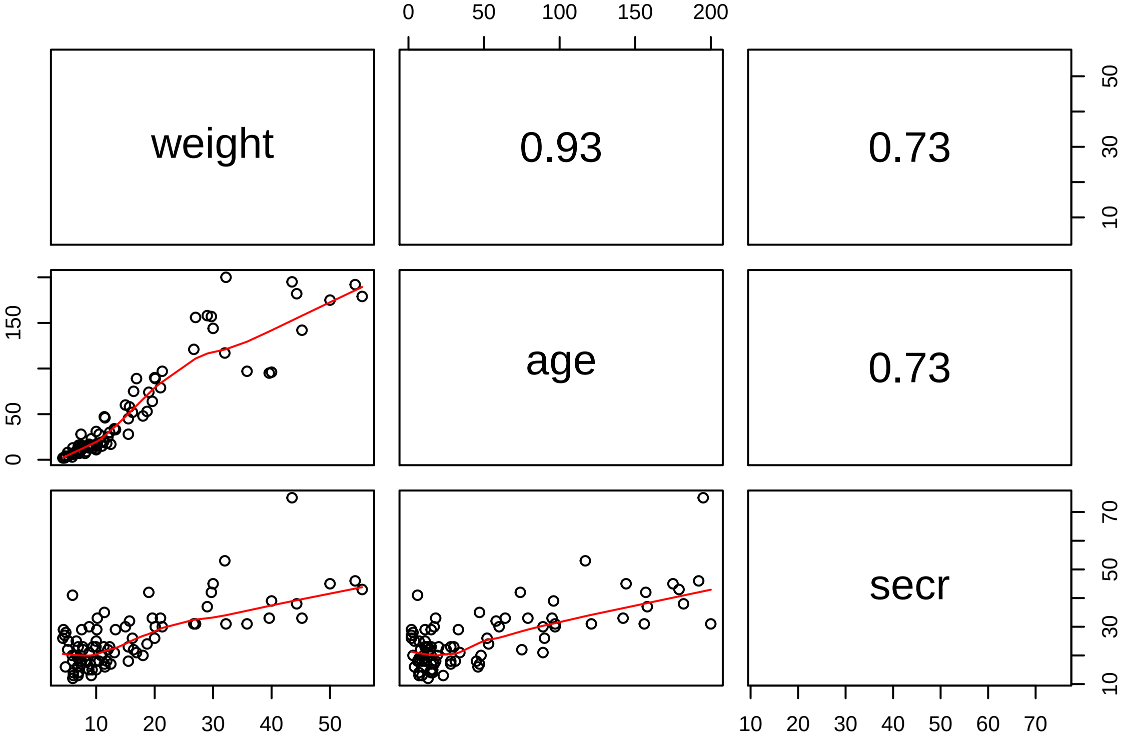

Figure S1: Correlations in covariates, plots in lower triangle, correlation coefficients in upper triangle

3 Further pharmacokinetic results

3.1 Treosulfan model basic goodness-of-fit

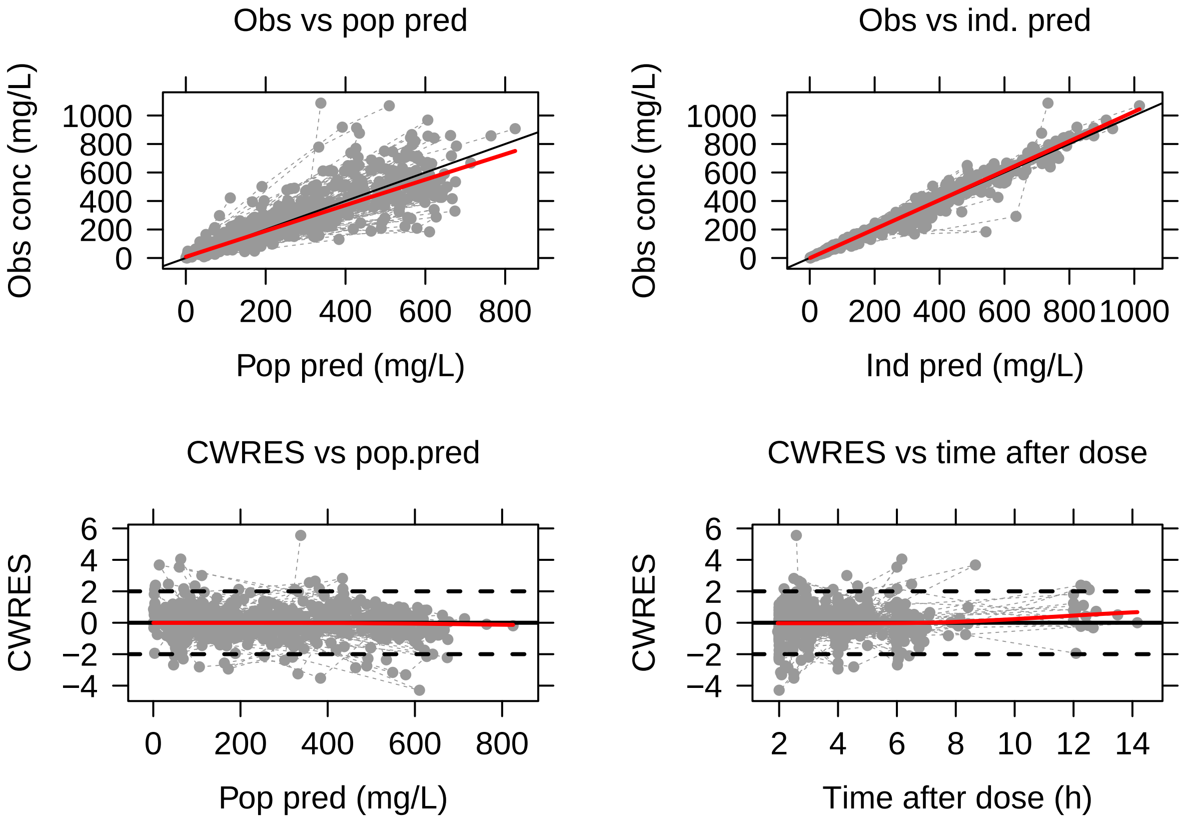

Figure S2: Population and individual predictions plotted against observed concentrations, and conditonal weighted residuals versus population prediction and time after dose

3.2 AUC and clearance relationship with age

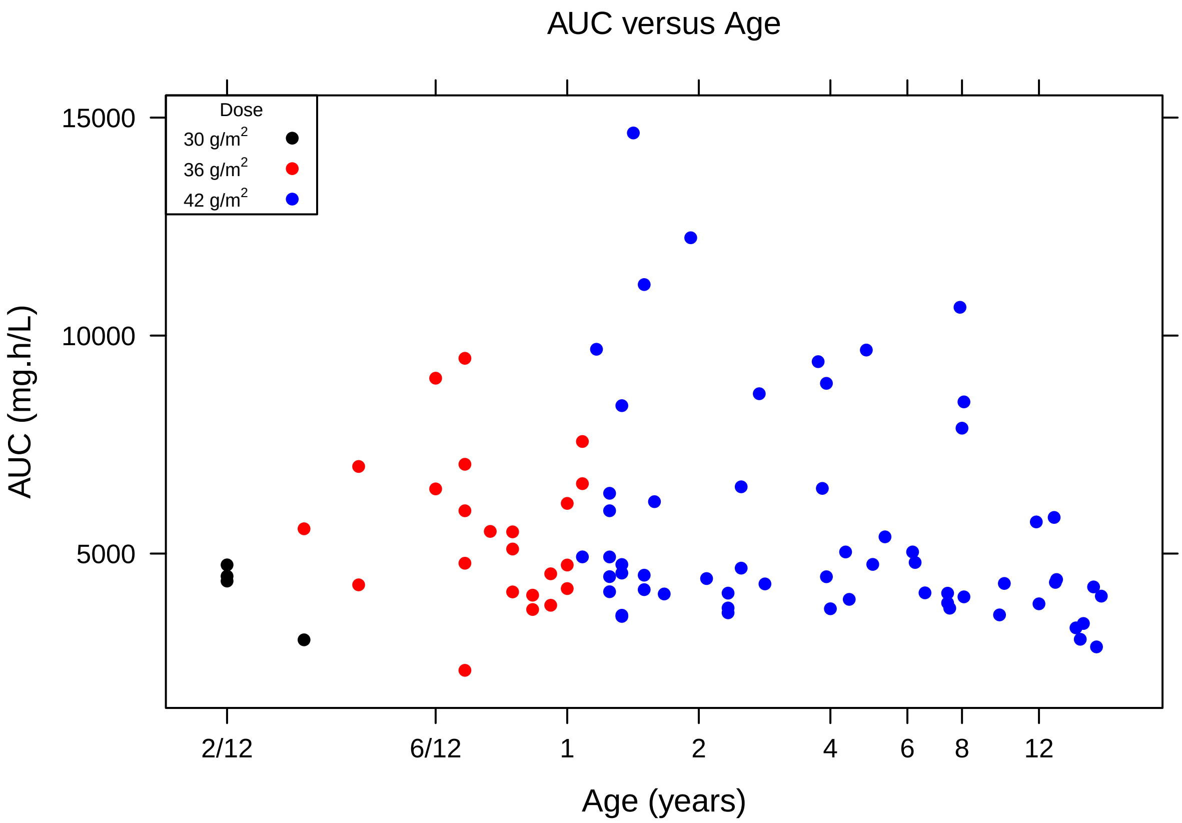

Figure S3: Relationship between AUC and age

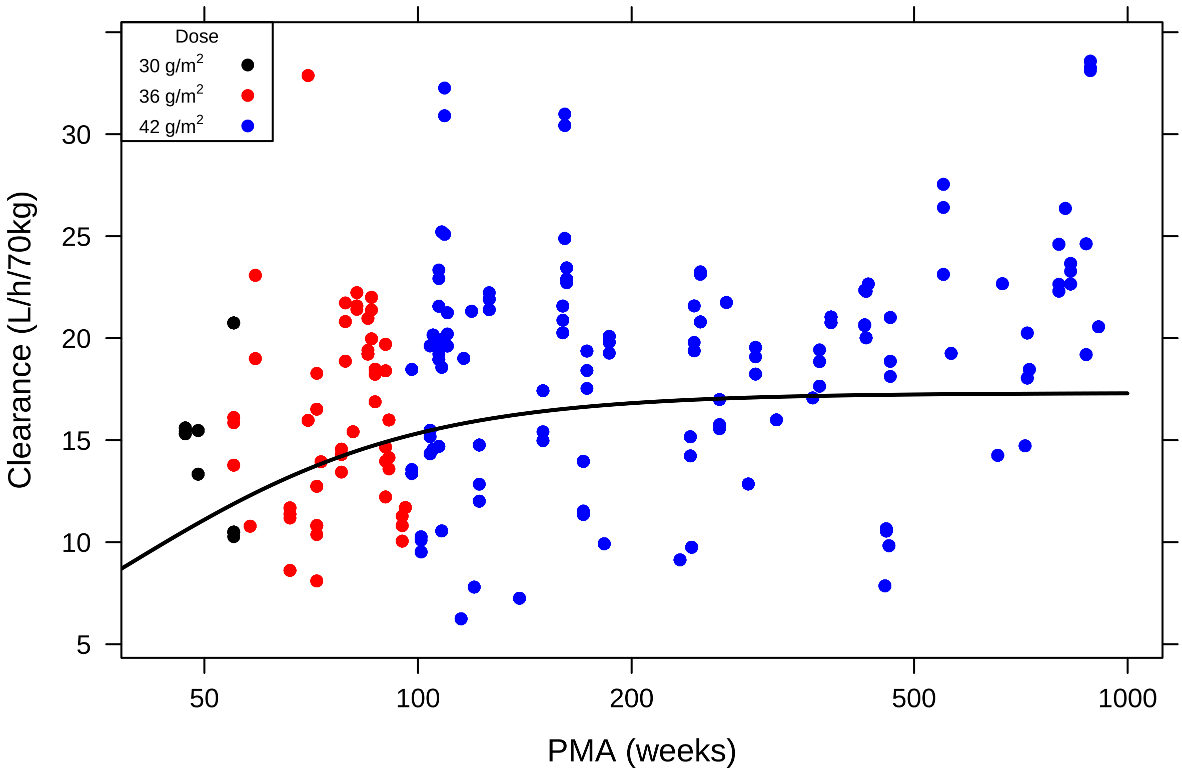

Figure S4: Relationship between size standardised clearance and age, line denotes model maturation function

4 Further Pharmacodynamic results

4.1 Graft versus Host Disease and AUC

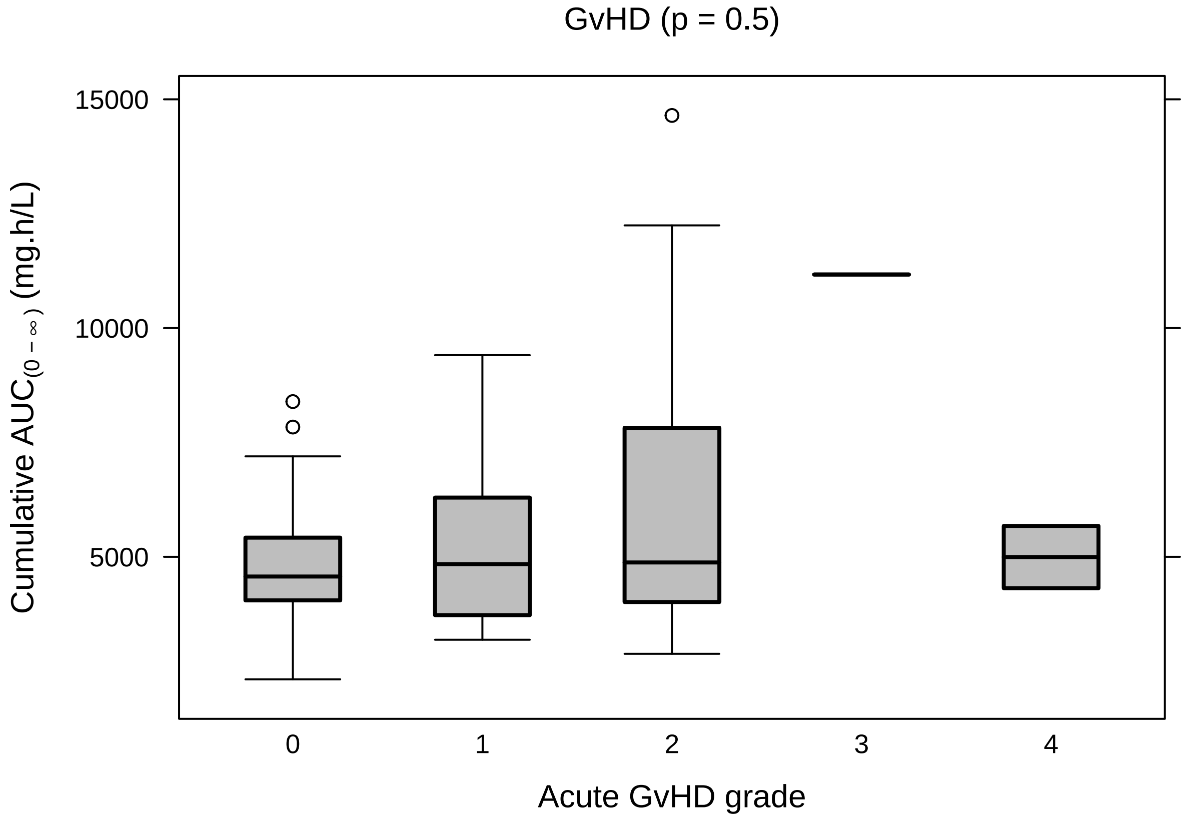

Figure S5: Relationship between acute GvHD and AUC with Kruskal Wallis test

4.2 Neutrophil recovery and AUC

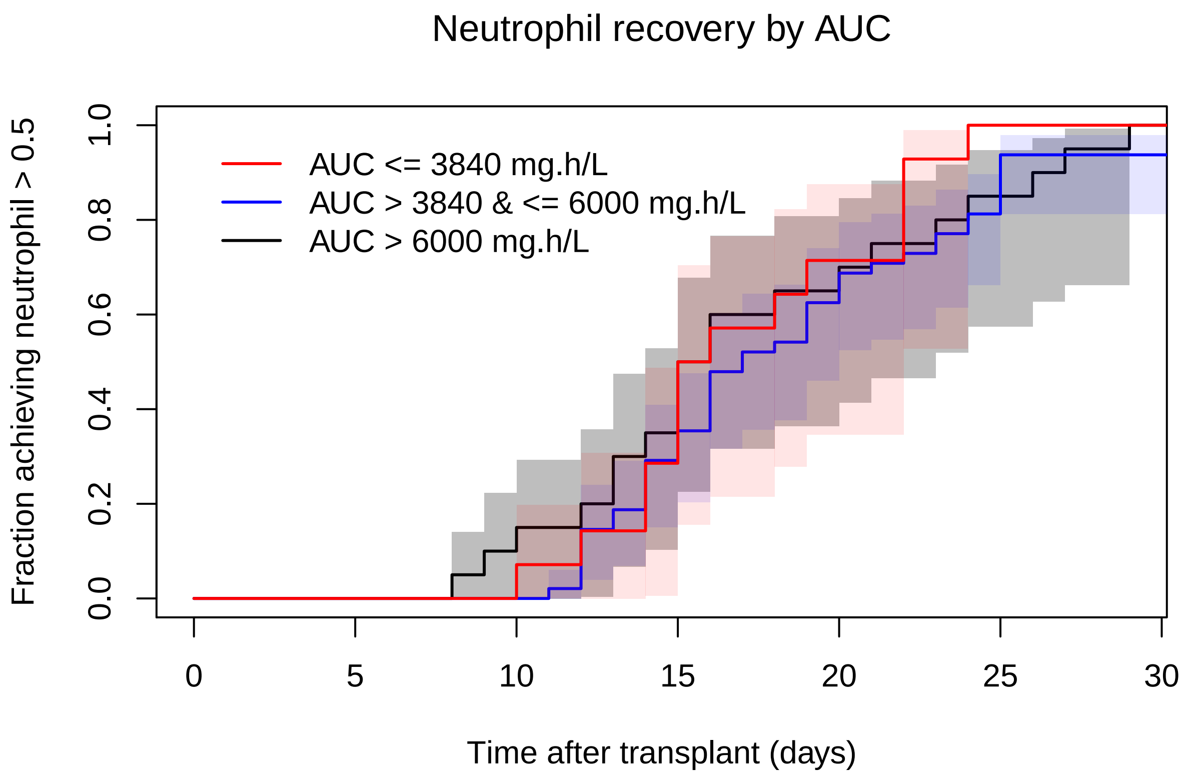

Figure S6: Time to neutrophil engraftment for patients below, in or above AUC target range

4.3 Platelet recovery and AUC

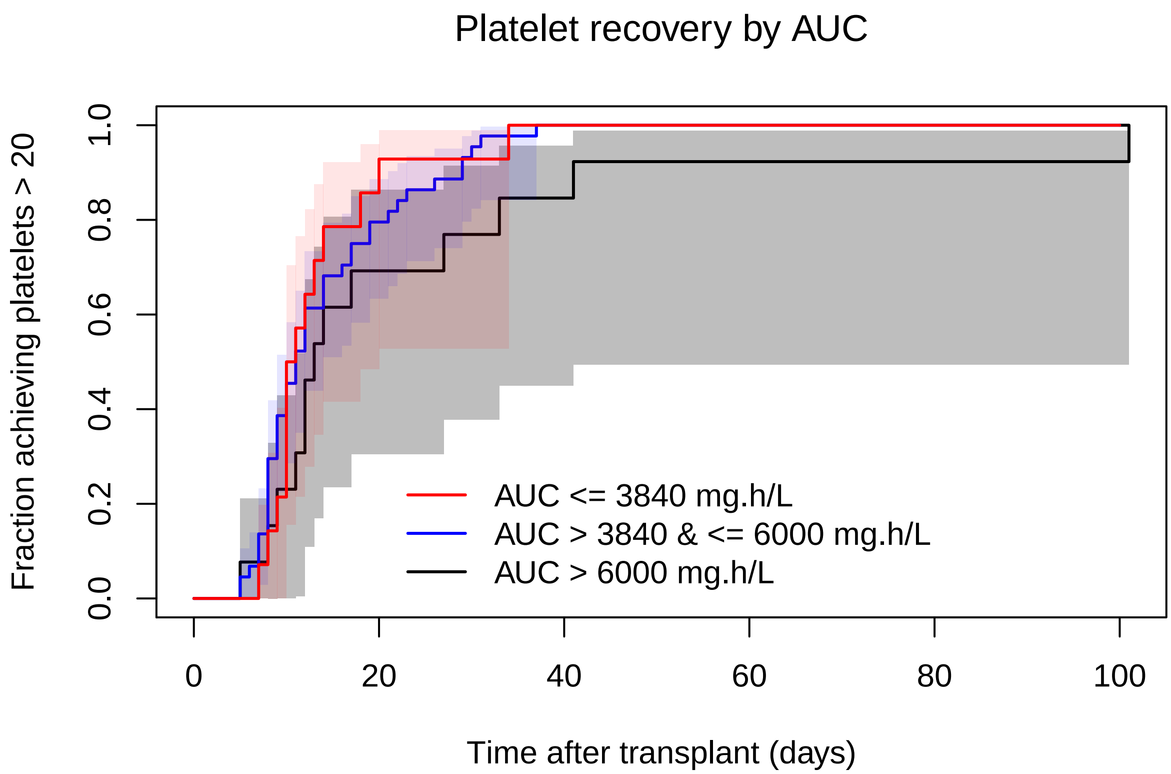

Figure S7: Time to platelet engraftment for patients below, in or above AUC target range

5 NONMEM pharmacokinetic model code

```
$PROBLEM    Treosulfan pk
$INPUT      ID TIME TAD AMT RATE DV EVID OCC BLQ WT AGEMO PMAW BSA
            DOSE AUCE HLE BILI ALT SECR SERO PH NEW COND SEX COHORT
            PIMM
$DATA       ./data/Treo_nonmem_180621.csv IGNORE=@
$SUBROUTINE ADVAN3 TRANS1
$PK
;--- TV param
 TVCL = THETA(1)
 TVV1 = THETA(2)
 TVQ2 = THETA(3)
 TVV2 = THETA(4)
 PM50 = THETA(5)
 HILL = THETA(6)
;--- MU param
 MU_1 = DLOG(TVCL)
 MU_2 = DLOG(TVV1)
 MU_3 = DLOG(TVQ2)
 MU_4 = DLOG(TVV2)
;--- Covariate functions
;--- Allometric and age scaling
 WTCL = (WT/70)**0.75
 WTV  = (WT/70)
 AGEF = 1/(1+(PM50/PMAW)**HILL)
;--- Mean expected creatinine for age
;--- (F. Ceriotti et al, Clinical Chemistry 54:3 559-566 (2008))
AGEY  = AGEMO/12
MSCR  = -2.37330-12.91367*DLOG(AGEY)+23.93581*AGEY**0.5
IF(AGEY>15)THEN
   IF(SEX==0)THEN
         MSCR = 9.5471*AGEY-87.847
   ELSE
         MSCR = 4.7137*AGEY-15.347
   ENDIF
ENDIF
;--- Creatinine covariate
 SCOV = (SECR / MSCR)**THETA(7)
;--- Between occasion variability
 BOV = 0
 IF(OCC==1) BOV = DEXP(ETA(5))
 IF(OCC==2) BOV = DEXP(ETA(6))
;--- Individual paramaters
 CL = WTCL * AGEF * BOV * SCOV * DEXP(MU_1 + ETA(1))
 V1 = WTV * DEXP(MU_2 + ETA(2))
 Q2 = WTCL * DEXP(MU_3 + ETA(3))
 V2 = WTV * DEXP(MU_4 + ETA(4))
;--- Rate constants
 K10 = CL/V1
 K   = K10
 K12 = Q2/V1
 K21 = Q2/V2
```

```
 BETA = 1/2*((K12+K21+K10)-SQRT((K12+K21+K10)**2-(4*K21*K10)))
 V   = V1
;
$ERROR
  IPRED = A(1) / V1
  Y     = IPRED * (1 + EPS(1)) + EPS(2)
  PROP  = SQRT(SIGMA(1,1))*IPRED
  ADD   = SQRT(SIGMA(2,2))
  SD    = SQRT(PROP*PROP + ADD*ADD) ; Standard deviation
  IRES  = DV - IPRED
  IWRES = IRES/SD
;--- Final estimates
$THETA  (0,17.3139) ; 1. CL
$THETA  (0,35.5482) ; 2. V1
$THETA  (0,9.36276) ; 3. Q2
$THETA  (0,9.89182) ; 4. V2
$THETA  (20,38.0144,80)  ; 5. PM50
$THETA  (0.5,2.11914,10) ; 6. HILL
$THETA  -0.30042         ; 7. SCOV
$OMEGA  BLOCK(2)
 0.09057
 0.108607 0.144922
$OMEGA  0  FIX
$OMEGA  0.18708
$OMEGA  BLOCK(1) 0.0206426
$OMEGA  BLOCK(1) SAME
$SIGMA  0.0182599
$SIGMA  0.920867
;--- Estimation method
$ESTIMATION METHOD=1 INTER MAXEVAL=0 PRINT=1
;$COVARIANCE
;--- Tables
$TABLE      ID AMT TIME TAD IPRED CWRES TIME IWRES NOPRINT ONEHEADER
            FILE=sdtab24
$TABLE      ID CL V1 Q2 V2 ETAS(1:5) BETA NOPRINT ONEHEADER
            FILE=patab24
$TABLE      ID TIME EVID OCC BLQ WT AGEMO PMAW BSA BILI ALT SECR SERO
            PH NEW COND RATE DOSE AUCE HLE COHORT PIMM SECR MSCR SECRN
            NOPRINT ONEHEADER FILE=cotab24
```
